# Relative Age Effect Is Widespread among Most Successful Youth, but Not in Senior Olympic Weightlifters

**DOI:** 10.5114/jhk/194499

**Published:** 2025-05-29

**Authors:** Eduard Bezuglov, Ryland Morgans, Elizaveta Kapralova, Evgeny Achkasov, Danila Telyshev, Olga Sadkovaya, Georgiy Malyakin

**Affiliations:** 1 Department of Sports Medicine and Medical Rehabilitation, Sechenov First Moscow State Medical University, Moscow, Russia.; 2 High performance sports laboratory, Sechenov First Moscow State Medical University, Moscow, Russia.; 3 N.A.Semashko Public Health and Healthcare Department, F.F. Erisman Institute of Public Health, Sechenov First Moscow State Medical University, Moscow, Russia.

**Keywords:** birth cohort, weight lifting, age groups, youth sports, age factors

## Abstract

The aim of the study was to investigate the prevalence of the relative age effect (RAE) among elite Olympic weightlifters in different age groups. The prevalence of RAE was studied among top 10 participants from the World Weightlifting championships for youth, junior and senior age groups and from the Olympic Games from 2009 to 2022. Birth dates of 3886 athletes were analyzed and further divided into four groups according to the birth quartile. Weight categories were grouped as lightweight, middleweight and heavyweight. The effect was found among lightweight and heavyweight girls, boys of all weight groups, lightweight and middleweight junior females and juniors of all weight groups. In the senior group, RAE was only present among heavyweight males. Differences in the prevalence of RAE between male and female weightlifters were statistically significant (p = 0.009). Differences in effect between youth and junior age groups were not significant (p = 0.24). The findings of this study demonstrate that RAE tends to be widespread among the best weightlifters of both sexes in youth and junior age groups, but disappears in most weight groups at the elite senior level.

## Introduction

Over the past decades, the focus of many coaches and researchers resided on studying the different patterns of athletes’ career development within the most popular competitive sports. The study of these patterns plays an important role in optimizing talent identification programs and may allow for the practical application of measures to retain the most gifted athletes. According to the meta-analysis by Güllich et al. (2023), only 11.8% of athletes who were successful at U17/18 competitions achieved the same success at the senior level, and 82% of successful senior athletes were not successful at the same age group competitions. Thus, successful junior athletes and successful senior athletes are largely two disparate populations (Güllich et al., 2023). This is further supported by the findings of Boccia et al. (2021a) which revealed the junior-to-senior transition rate at 16 and 18 years of age was only 6% and 12% in male and 16% and 24% in female elite throwers, respectively. Such career patterns of successful athletes in different age groups can be related to several socio-cultural factors and specific features of particular sports (age at which the athlete began their regular training, the level of competition, popularity of the sport) (Hancock et al., 2013). One of the most studied factors directly affecting athletic success in adolescence is the relative age effect (RAE), which was first described almost 40 years ago (Barnsley et al., 1985).

The RAE is the over-representation of athletes born closer to the date used to divide athletes into specific age groups (Wattie et al., 2008). Given the fact that the vast majority of sports organizations divide athletes into groups according to the January 1–December 31 time period, RAE most often consists of over-representation of athletes born in the first quarter ("early-born") and under-representation of athletes born in the fourth quarter ("late-born").

According to available data RAE is prevalent in young men and women in sports with early specialization and a high level of competition such as soccer and ice hockey (Fumarco et al., 2017; García-Rubio et al., 2022; Morgans et al., 2024). Garcia-Rubio et al. (2022) established that RAE was present in U17 to U21 Spanish youth national teams. Furthermore, youth female soccer players born in the first birth quartile were two times more likely to be selected to the national team compared to those born in the fourth birth quartile. In the U17 team, goalkeepers, defenders, and midfielders of the first birth quartile were overrepresented (Brustio et al., 2023). RAE has also been identified in a number of combat sports. A study on international judo athletes by Fukuda et al. (2023) reported a higher prevalence of RAE in males than females, and cadets and juniors compared to seniors. RAE was present in heavyweight and middleweight categories in senior and junior males, while for females in cadet heavyweights only. In elite Brazilian karate, RAE occurred in U14 and U16 categories, being higher in males. RAE was similar between the sexes in the kumite modality. However, there was no RAE for the kata modality in karate and for the taekwondo categories (De Almeida-Neto et al., 2023). The widespread prevalence of RAE in female sports among pre-adolescent and adolescent age groups was also demonstrated in a systematic review conducted by Smith et al. (2018).

One of the reasons for the widespread RAE prevalence in sport is physical and psychological developmental advantage that “early-born” athletes may have due to their higher biological maturity status (Hill et al., 2020; Wattie et al., 2015). In addition, the implications of RAE in younger age groups are undoubtedly perpetuated through perceptions of athlete competence, including athletes’ perceptions of themselves (i.e., Galatea effect), their coaches (i.e., Pygmalion effect), and their parents (i.e., Matthew effect) (Merton, 1968; Zanna et al., 1975). Initially, parents may influence RAE by encouraging more frequently the relatively older athletes to take up sports (i.e., Matthew effect). Meanwhile, coaches might place greater expectations (e.g., more attention during the training sessions) on relatively older athletes and consequently advantage them (i.e., Pygmalion effect). Finally, older athletes, due to the higher expectation of parents and coaches, may increase their self-efficacy (e.g., perceive themselves as being more gifted) and be more motivated to work harder to meet expectations (Brustio et al., 2022; Hancock et al., 2013).

In connection with the above data, it is logical to assume that the prevalence of RAE will be significant not only in the most competitive team and individual sports with early specialization, but also in sports, where the age at which athletes begin their regular training is higher, and the key quality for success is strength. One such a sport is weightlifting, which is most often started at the age of 10–12 (Dvorkin, 1992; Pierce et al., 1999, 2022). To date, the research of RAE in weightlifting in peer-reviewed journals is scare. However, in previous studies, participants were only senior Olympic competitors or youth and senior weightlifters at the national level (Erdaği and Işik, 2023; Kollars et al., 2021; Tüfekçi et al., 2022). For example, the study by Kollars et al. (2021) analyzed the prevalence of RAE among Olympic participants. The effect was present in lightweight, middleweight, heavyweight men and lightweight women. However, that study only analyzed birth dates of senior athletes and did not include intergroup comparisons. This does not allow us to draw conclusions about the prevalence of RAE among the most successful athletes of both sexes in different age groups (Kollars et al., 2021). The absence of these data compromises the development pathways for late-born athletes, who may face early career discrimination in terms of short- and mid-term sporting success, not due to a lack of talent, but because of their lower degree of biological maturation.

Thus, the aim of this study was to investigate the prevalence of RAE among elite Olympic weightlifters in different age groups. The study tested the hypothesis that the prevalence of RAE would be most present among young athletes and would start declining among the senior group, but there would be still more “early-born” athletes than “late-born” among them.

## Methods

### Participants

For the purposes of this study, we defined “most successful athletes” as those who achieved a place within the top 10 in the largest international competitions for their respective age groups, which included the World Weightlifting Championships for youth (age 13–17), juniors (age 15–20), and seniors (typically aged above 20, however, the entry age being 15), as well as the Olympics for seniors (Weightlifting age groups, accessed on 10 February 2023). The final analysis of the RAE prevalence included participants’ data obtained from a total of 10 World Youth Championships, 13 World Junior Championships, 11 World Senior Championships, and three Olympic Games held between 2009 and 2022 (IWF event results, accessed on 15 February 2023). The beginning of the first time period (2009) was selected as it coincided with the First World Youth Weightlifting Championship.

Weight categories were grouped as per the classification previously described by Gottwald et al. (2021) and adjusted to the new weight categories implemented by the IWF in 2018 (Table 1) (Gottwald et al., 2021).

**Table 1:** Breakdown of weight groups and categories.

Sex and time period	Age group	Weight group (kg)		
		Lightweight	Middleweight	Heavyweight
Female 1998–2017	Youth	44, 48	53, 58, 63	69, 69+
	Junior and Senior	48, 53, 58	63, 69	75, 75+
Female 2018–present	Youth	40, 45, 49	55, 59, 64	71, 76, 81, 81+
	Junior and Senior	45, 49, 55	59, 64, 71	76, 81, 87, 87+
Male 1998–2017	Youth	50, 56	62, 69, 77	85, 85+
	Junior and Senior	56, 62	69, 77, 85, 94	105, 105+
Male 2018–present	Youth	49, 55	61, 67, 73, 81, 89, 96	102, 102+
	Junior and Senior	55, 61	67, 73, 81, 89, 96	102, 109, 109+

A total of 3886 athletes' birth dates were analyzed and sub-divided into four age groups according to the birth quartile:

- first quarter (Q1) January–March, “early-born”,
- second quarter (Q2) April–June,
- third quarter (Q3) July–September,
- fourth quarter (Q4) October–December, “late-born”.

For each one of these birthdate groups, the relative sample size was calculated using the formula “100 x the number of the date of birth group/total number”. RAE was defined as a higher relative sample size in the first quarter compared to other quarters by the date of birth (Bezuglov et al., 2020).

The study was performed in accordance with the Declaration of Helsinki. This study was approved by the Ethics Committee of the Sechenov First Moscow State Medical University (approval code: N 22-11; approval date: 09 December 2021). The data were obtained from open access sources, therefore no informed consent for study participation was necessary.

### Design and Procedures

The study is of a cross-sectional observational design (level of evidence 3). An analysis of the results achieved by the most successful male and female weightlifters from various age groups and weight categories was conducted using competition protocols obtained from the official International Weightlifting Federation (IWF) website (IWF event results, accessed on 15 February 2023).

The prevalence of RAE was analyzed (1) across weight groups, (2) across sexes, (3) across age groups, and (4) separately for males and females across different weight and age groups. In addition, a list of top-3 weight groups with the most and least pronounced RAE was compiled.

Athletes belonged to the age and weight groups in which they were at the time of a particular competition. One athlete might only enter each age group once, i.e., if the athlete won the World Senior Championships several times, only one of the results in particular age group was included in the analysis. In the event of the age overlap, if a youth or junior athlete achieved a place within the top 10 in a World Championship in the senior group or in the Olympic Games, the result was included in the age group of the competition. For instance, if an 18-year-old athlete competed in a senior event, he/she was classified as a senior. If the athlete participated in a junior level competition, they were assigned to the junior group.

### Statistical Analyses

The data were analyzed using the statistical package IBM SPSS v. 26.0, Armonk, New York; USA. Frequency analysis was used to describe the RAE. A Chi-square with an arbitrary number of cells was used to compare the prevalence of RAE among the analyzed groups by age (youth, junior, senior), sex and weight groups (1: lightweight, 2: middleweight, 3: heavyweight). Odds ratios and 95% confidence intervals were calculated in the presence of statistically significant results in 2 x 2 tables. Results were considered significant at *p* < 0.05. Effect size (Cramer`s V) was calculated for statistically significant results. Effects size descriptors were as follows: ≤ 0.2 = large, ≤ 0.6 and > 0.2 = medium, > 0.6 = high (Cramér, 1999).

## Results

**Figure 1:**
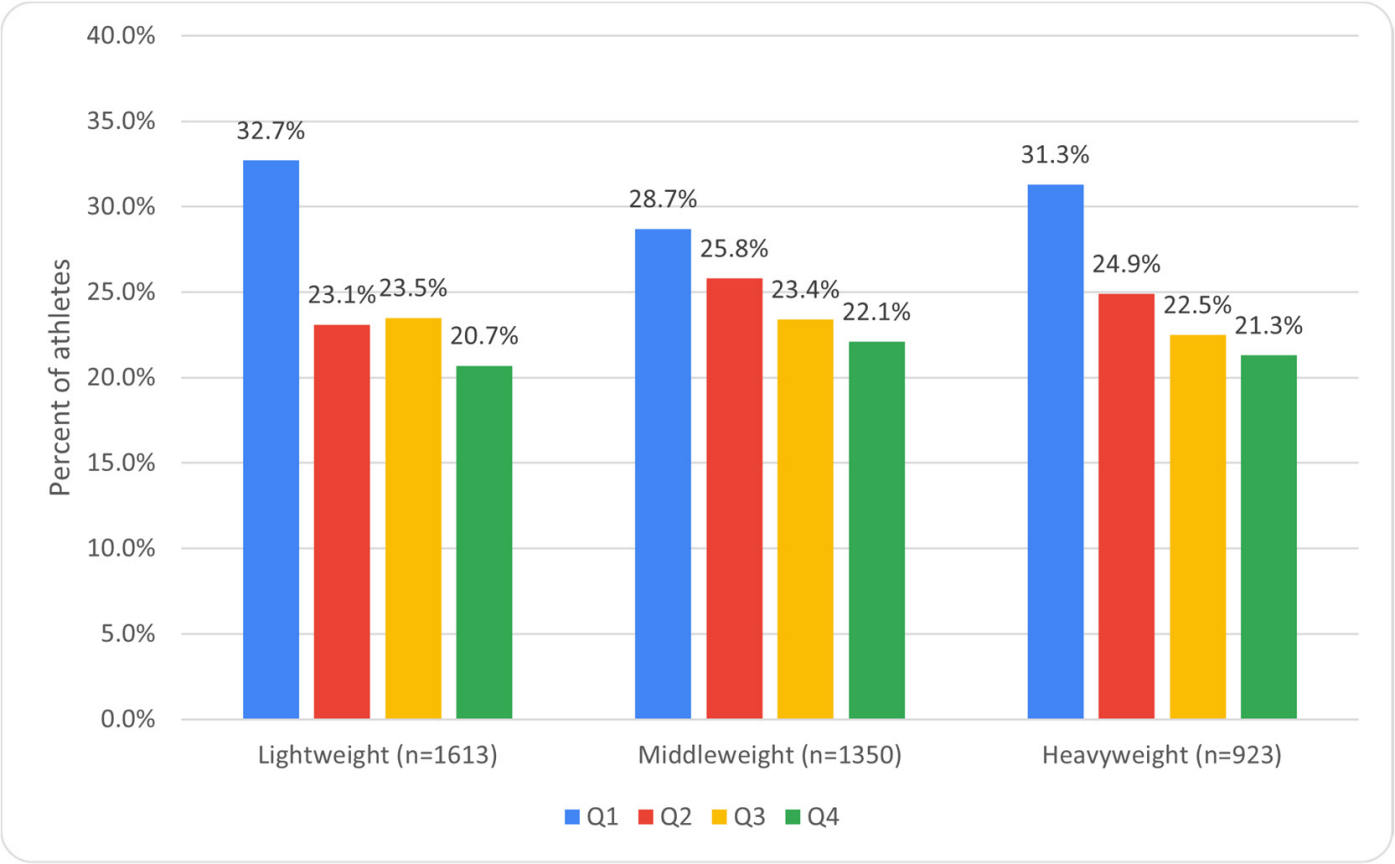
Prevalence of RAE among the most successful weightlifters of different age and weight groups with no regard to sex.

When analyzing the prevalence of RAE among athletes of both sexes, its presence was observed in all three weight groups. Furthermore, no significant difference was found in the effect size among these groups (Figure 1).

Differences in the prevalence of RAE between male and female weightlifters were statistically significant (*p* = 0.009, ES = 0.06). Such significance was found due to more Q1 athletes (*p* = 0.002, OR 1.8, 1.06–3.03, ES = 0.06) and fewer Q4 athletes (*p* = 0.045, OR 0.73, 0.53–0.99, ES = 0.03) among males in pairwise comparisons. No significant difference was found when comparing the number of athletes in the other birth quartiles (Table 2).

**Table 2:** Distribution by the birth quartile of the most successful male and female weightlifters.

Age group	Sex	Total n	Weight group	n	Q1%	Q2%	Q3%	Q4%
Youth	Female	633	light	332	32.8	23.3	26.2	17.7
			middle	182	23.6	30.8	25.8	19.8
			heavy	119	31.9	26.0	21.8	20.3
	Male	721	light	341	39.9	23.2	20.8	16.1
			middle	281	35.2	22.1	22.8	19.9
			heavy	99	40.4	27.3	20.2	12.1
Junior	Female	685	light	266	30.8	24.8	22.9	21.5
			middle	198	32.3	22.7	21.7	23.3
			heavy	221	26.2	24.4	22.6	26.8
	Male	808	light	288	34.4	20.1	22.6	22.9
			middle	319	29.5	29.1	21.6	19.8
			heavy	201	36.8	20.9	25.9	16.4
Senior	Female	511	light	199	25.1	28.1	23.6	23.2
			middle	157	22.3	26.7	20.4	30.6
			heavy	155	22.6	30.3	21.9	25.2
	Male	527	light	186	28.0	21.5	25.8	24.7
			middle	213	24.4	23.5	28.6	23.5
			heavy	128	34.4	22.7	20.3	22.6

**Figure 2:**
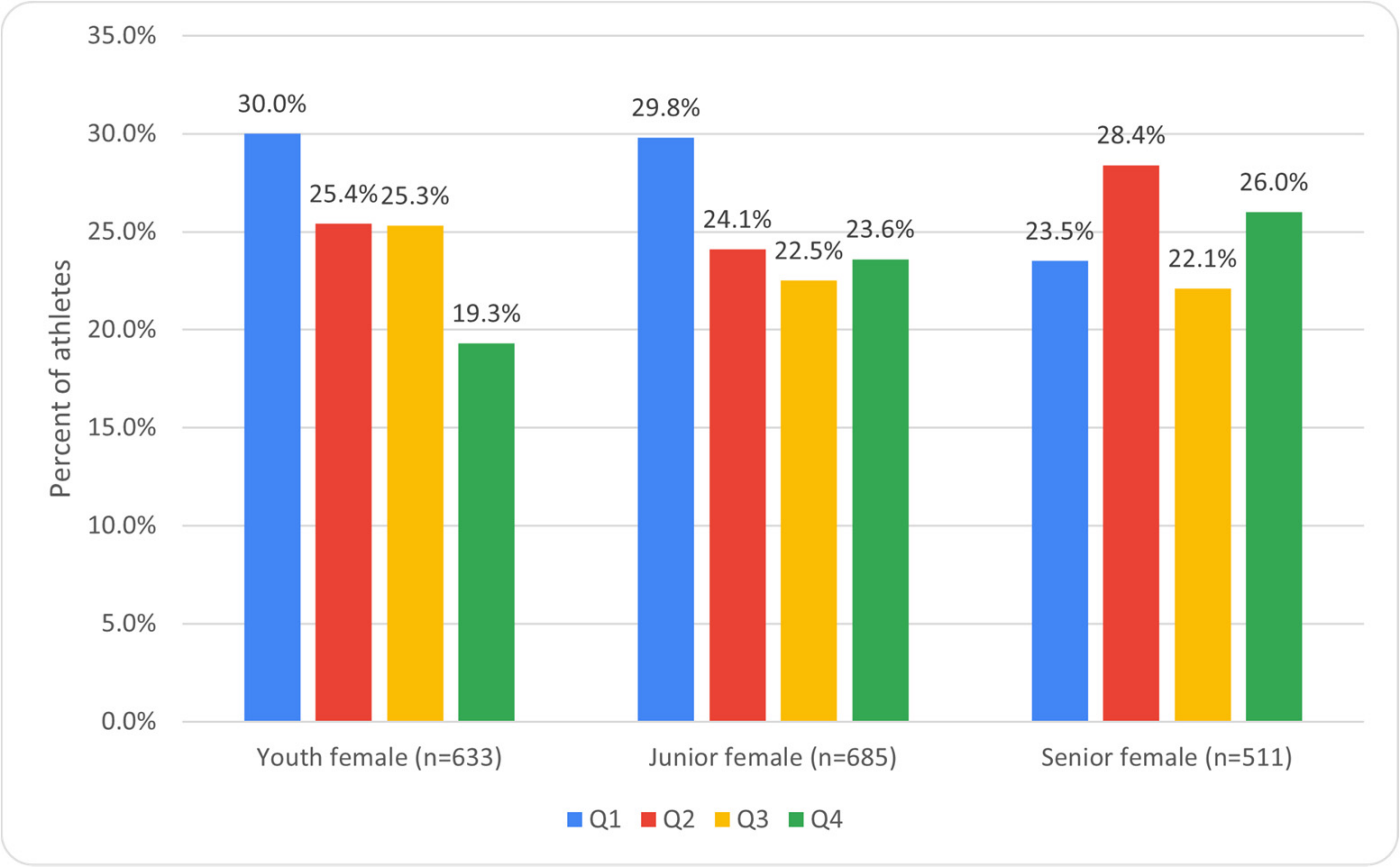
Prevalence of RAE among the most successful female weightlifters of different ages with no regard to weight groups.

When analyzing the dates of birth of athletes of the same sex who competed in different age groups, significant differences were found in the number of Q1 and Q4 female athletes among youth compared to senior female athletes (*p* = 0.007, ES = 0.13). The number of Q1 female athletes among juniors was also significantly higher than in the senior group (*p* = 0.016, OR 1.38, 1.06–1.80, ES = 0.07). Differences in RAE between youth and junior age groups were not significant (Figure 2).

**Figure 3:**
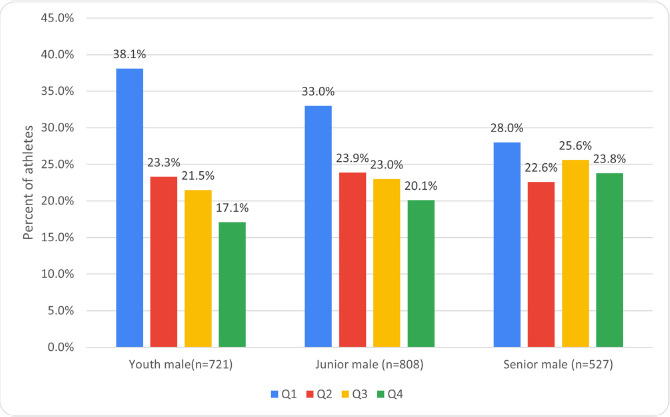
Prevalence of RAE among the most successful male weightlifters of different ages with no regard to weight groups.

It was also found that for males there were significantly more Q1 athletes (*p* < 0.001, OR 1.58, 1.24–2.01, ES = 0.10) and significantly fewer Q4 athletes (*p* = 0.004, OR 0.66 0.5–0.87, ES = 0.08) among youth than seniors. Differences between juniors and seniors were not significant. Differences in RAE between youth and junior athletes were not significant. However, there was a higher proportion of Q1 athletes among youth compared to juniors (*p* = 0.038, OR 1.25, 1.01–1.54, ES = 0.05) (Figure 3).

### Youth Age Group

The analysis revealed a high prevalence of RAE among athletes of both sexes (Figures 2 and 3). RAE was most pronounced among male athletes (*p* = 0.018, ES = 0.08) due to the higher number of Q1 athletes among them (*p* = 0.002, OR 1.44, 1.15–1.80, ES = 0.08). When dividing all male and female athletes by weight groups, the prevalence of RAE was observed among lightweight and heavyweight girls, and among boys of all weight groups (Table 2).

There were no significant differences in the prevalence of RAE among male and female athletes within any weight group.

### Junior Age Group

The analysis revealed a high prevalence of RAE among athletes of both sexes (Figures 2 and 3). Differences in RAE were not significant among both sexes. When dividing male and female athletes by weight groups, the prevalence of RAE was observed among lightweight and middleweight junior females as well as among junior males in all weight groups (Table 2).

When analyzing the prevalence of RAE among junior male and female athletes across different weight groups, no significant differences were found between the groups.

Significant difference was found in the prevalence of RAE between athletes of different sexes among heavyweights (*p* = 0.02, ES = 0.11). In particular, there were more Q1 athletes (*p* = 0.02, OR 1.64, 1.08–2.48, ES = 0.11) and fewer Q4 athletes (*p* = 0.011, OR 0.54 (0.33–0.87, ES = 0.12) among males.

### Senior Age Group

Analysis showed no RAE among either senior male or female weightlifters (Figures 2 and 3). Additionally, differences in RAE were not significant between the sexes. RAE prevalence was observed only among heavyweight males when athletes were categorized by the weight group (Table 2).

Analysis of RAE prevalence among male and female athletes across weight groups revealed a higher number of Q1 heavyweight males compared to middleweight males (*p* = 0.048, OR = 0.62, 0.38–1.00, ES = 0.11). No significant differences were found in other weight group comparisons (Table 2).

### Groups of Athletes with the Most and Least Pronounced RAE

The analysis demonstrated that the highest prevalence of RAE was observed among male youths in the lightweight and heavyweight groups, and juniors in the heavyweight group. The effect was least pronounced among senior females in the middleweight and heavyweight groups, and among junior females in the heavyweight group.

## Discussion

This study demonstrated that RAE was widespread among the best weightlifters of both sexes, competing in the youth and junior age groups in almost all weight groups. At the senior level, however, this effect was only pronounced among male athletes competing in the heavyweight group. This study was one of the first to examine the prevalence of RAE among top weightlifters of all age groups. In previous studies, participants were only senior Olympic competitors or youth and senior weightlifters at the national level (Erdaği and Işik, 2023; Kollars et al., 2021; Tüfekçi et al., 2022). In those studies, the effect was common in most weight categories and more pronounced in male athletes. Given the lack of research on this topic among weightlifters, it is not possible to conduct a meaningful comparative analysis of the prevalence of RAE at different time periods among weightlifters from countries with different levels of competition. However, even the available data suggest that in sports with relatively late specialization (compared to soccer and ice hockey), the prevalence of RAE may be widespread. Considering weightlifting, the most likely reason for this may be the dependence of performance on athlete strength, which may be particularly true in the early specialization stages when differences in exercise technique may not be meaningful due to the overall small length of training experience.

This can be confirmed by the data of studies involving elite young track and field athletes and judokas, i.e., representatives of sports where strength is also significant. For example, Fukuda (2015) found a high prevalence of RAE in elite young judo athletes of different age groups, sex and weight categories (medalists of cadet and junior judo world championships) in most weight categories. In terms of track and field, there are a number of studies supporting the presence of this effect among the youngest competitive-level track and field athletes (Bezuglov et al., 2022b, 2023; Gundersen et al., 2022; Kearney et al., 2018). In addition, RAE is present in throwing, where strength plays a key role. The effect has been observed among elite throwers at the junior and even the senior level (Boccia et al., 2021a).

Given that strength development is significantly associated with serum testosterone concentrations, it should not be surprising that early-born and early-maturing athletes (i.e., those with faster growth spurts) would have a potential advantage over their peers up to the age of plateau in serum testosterone concentrations, 14 and 17 years of age for girls and boys, respectively (Senefeld et al., 2020). The key role of testosterone in the achievement of athletic success in weightlifting can be indirectly confirmed by the widespread use of various anabolic agents as doping in this sport. According to Kolliari-Turner et al. (2021) between 2008 and 2019, of 565 sanctions against weightlifters, 82% involved the detection of exogenous metabolites of anabolic androgenic steroids and markers indicating endogenous use.

To explain the disappearance of RAE among the best senior weightlifters, we can look to a study by Bush et al. (2021). Those authors surveyed over 140 elite senior weightlifters and found that less than 25% began specializing before the age of 18, and the vast majority of them (over 75%) did not specialize until the age of 21. However, the incidence of injury was higher among athletes with earlier specialization and the majority of athletes (about 70%) believed that specialization in the youth age group was not necessary to achieve elite status (Bush et al., 2021). That is, it could be argued that many successful weightlifters at the senior level began their specialization when older chronological age no longer played a role and were less susceptible to injuries that can have a negative impact on success at any stage of a sporting career.

It should also be noted that the findings indicate the prevalence of RAE in most weight groups among female athletes. Although not all previous studies have reported similar results, they support observations of Smith et al. (2018) whose meta-analysis demonstrated that the magnitude of RAE was higher in team and individual sports involving high physiological exertion.

One limitation of this study is the lack of comparison of the RAE prevalence among most successful weightlifters relative to their less successful peers. Moreover, the study is limited by the lack of data on the career trajectory of the most successful weightlifters at the youth level. Similar studies have previously been conducted for example in track and field and have demonstrated that only a small proportion of athletes who are successful at the youth level also succeed at the senior level (Bezuglov et al., 2022a; Boccia et al., 2021b). The cross-sectional design is another limitation of the study (Huebner and Perperoglou, 2019, 2020). A longitudinal study that follows athletes through various phases of their development could provide a more detailed understanding of the dynamics of RAE and its long-term impact on sports success. Furthermore, this study does not include detailed information on the onset of athletes' specialization, their training, and possible injuries. Moreover, the study does not consider socio-cultural factors that may influence RAE, such as family support, access to training and resources, and motivation. These factors can significantly affect athletes' development and success and could provide an additional context for interpreting RAE results.

Future studies should be longitudinal and provide more detailed analysis and comparison with less successful athletes. Furthermore, considering RAE in different geographical areas and at various competition levels could provide insights into how RAE manifests in different contexts. It could also help identify specific strategies for mitigating RAE that are effective in certain environments.

## Practical Implications

Coaches should be aware that early-born athletes may have a short-term advantage at the youth and junior levels due to greater physical strength and experience. However, at the elite senior level, this advantage could disappear in many weight categories as athletes reach their full maturity. In addition, given the specifics of the sport, many athletes successful at the senior level start training and competing after puberty. Consequently, it can be concluded that a more strict selection among early-born athletes can be a good strategy for raising top performers, yet it is equally important to not only allow, but encourage late-born weightlifters to train and participate in competitions. Considering these data will help retain many weightlifting athletes who might otherwise end their careers prematurely due to a lack of performance success.

## Conclusions

The findings of this study demonstrate that RAE tends to be widespread among the best weightlifters of both sexes in youth and junior age groups, but disappears in most weight groups at the elite senior level.
